# Evidence of individual differences in the long-term social, psychological, and cognitive consequences of child maltreatment

**DOI:** 10.1186/s13034-022-00524-4

**Published:** 2022-11-23

**Authors:** Rosa S. Wong, Keith T. S. Tung, Ko Ling Chan, Wilfred H. S. Wong, Hing Wai Tsang, Clare H. Y. Chow, Gilbert T. Chua, Winnie W. Y. Tso, Jason C. Yam, Ian C. K. Wong, Patrick lp

**Affiliations:** 1 Department of Paediatrics and Adolescent Medicine, The University of Hong Kong, Hong Kong, SAR China; 2 Department of Pharmacology and Pharmacy, The University of Hong Kong, Hong Kong, SAR China; 3 Department of Applied Social Sciences, The Hong Kong Polytechnic University, Hong Kong, SAR, China; 4 State Key Laboratory of Brain and Cognitive Sciences, The University of Hong Kong, Hong Kong, SAR China; 5 Department of Ophthalmology and Visual Sciences, The Chinese University of Hong Kong, Hong Kong, SAR, China; 6 Research Department of Practice and Policy, UCL School of Pharmacy, London, UK

**Keywords:** Child maltreatment, Cytokine, Inflammation, Family support, Stress, Adulthood

## Abstract

**Background:**

The prevalence and consequences of child maltreatment are alarming, but evidence from studies with long follow-up intervals are limited. This study examined the long-term consequences of child maltreatment in relation to age of onset and follow-up interval.

**Methods:**

The exposed group comprised 63 individuals (aged 13–34 years) with a first-time diagnosis of child maltreatment between 2001 and 2010, whereas the unexposed group comprised 63 individuals who were matched upon gender, age of onset, follow-up period, and poverty status at the index hospital admission but had no medical records of maltreatment in Hong Kong. The participants completed a set of questionnaires on executive functions and mental health and provided blood samples for measurement of IL-6 and IL-10 levels during a health assessment session.

**Results:**

Compared with the unexposed group, the exposed group reported poorer maternal care during childhood (β = −4.64, p < 0.001) and had lower family support (β = −2.97, p = 0.010) and higher inflammatory responses (IL-6: β = 0.15, p = 0.001; IL-10: β = 0.11, p = 0.011) at follow-up. Additionally, the associations of childhood maltreatment exposure with family support and maternal care differed by age of onset and the length of time since exposure.

**Conclusions:**

This matched cohort study highlights childhood maltreatment as a risk factor for systemic inflammation and an indicator of suboptimal social environment, both of which could persist over a long period of time.

**Supplementary Information:**

The online version contains supplementary material available at 10.1186/s13034-022-00524-4.

## Background

Child maltreatment, including all types of physical and/or emotional ill-treatment, sexual abuse, neglect, negligence and commercial or other exploitation [1], is a prevalent problem in many parts of the world including Chinese societies [2, 3]. In the absence of proper interventions, childhood maltreatment experiences can lead to long-term physical and mental health problems. Emerging results from Western studies with long-term follow-up have demonstrated the enduring effects of childhood maltreatment experiences. For example, a prospective study of 3778 mother and child pairs in Australia found that a history of substantiated child maltreatment was strongly associated with youth self-reported depressive symptoms and internalizing and externalizing problems at approximately 21 years of age [4]. However, most evidence in Chinese societies are drawn from cross-sectional research or longitudinal research with a relatively short follow-up period [5]. As the ecological systems theory suggests [6], culture is one of the factors that can contribute to differences in reported consequences of childhood events between populations. In order to better elucidate the complex nature of child maltreatment and its consequences in different cultures, more studies with long-term follow-up, particularly in non-Western cultures, are needed [7].

In addition, despite much evidence on stress-related diagnoses or questionnaire-assessed psychological distress, little work has been done to examine physiological responses to childhood maltreatment exposure. Some data from Western populations suggests that proinflammatory cytokines such as IL-6 and IL-10 levels are higher among individuals with childhood trauma compared with those not exposed, but findings of this kind are mainly obtained from patient samples [8, 9]. More studies should be conducted to explore the association between childhood maltreatment and inflammation level in non-patient samples. Notably, individuals from different backgrounds may give differential responses to the same exposure. Evidence from both patient and non-patient samples will enhance our understanding of whether the observed physiological responses associated with child maltreatment can be generalized to all populations.

The consequences of child maltreatment are also affected by individual-level factors such as age of maltreatment onset and resilience [10], but previous findings about age of maltreatment onset have been limited by their single focus on emotional problems [11], gender-specific addictive behavior [12], or patient samples [13]. Some studies suggest that compared to recent traumas, early trauma memories, particularly for those experiences that are interpersonal in nature including child maltreatment events, can impair more functional domains [14]. Building upon these earlier findings, it would be interesting to explore the perception of adults with a history of childhood maltreatment toward their current and past home environment, particularly with respect to parental care and family support. Lastly, while a growing body of evidence points to the association between child maltreatment and symptoms of attention deficit hyperactivity disorder (ADHD) in childhood and adolescence [15], little is known about the association between maltreatment experiences in childhood and executive functions in adulthood.

To address these knowledge gaps, we conducted this matched cohort study with a long follow-up period ranging from 10 to 20 years in a predominantly Chinese society. Its primary objective was to compare the long-term social, psychological, and cognitive outcomes of individuals with a history of childhood maltreatment substantiated through hospital records and those without such exposure. The exposed and unexposed groups were matched based on gender, age of onset, follow-up duration, and status of poverty on the day of hospital admission. We also explored whether child maltreatment effects will differ by age of onset and follow-up duration.

## Methods

### Study design and participants

This is a matched cohort study involving 236 Hong Kong Chinese individuals identified from an existing research database containing their contact information. The exposed cohort comprised 85 individuals who were admitted to the pediatric ward of a local teaching hospital under the age of 18 years due to one type or multiple types of maltreatment defined by the International Classification of Diseases (ICD-9-CM) codes (955.50-955.59 or E967.0-E967.9) for the first time during 2001–2010 as recorded in the Hong Kong Clinical Data Analysis and Reporting System (CDARS), which is an electronic database used by the public healthcare system in Hong Kong. All these maltreatment cases were confirmed through a detailed investigation conducted by a multidisciplinary team, which has been described in a previous study [16]. The unexposed cohort comprised 151 patients who were admitted to the same hospital under the age of 18 years due to common cold, bronchitis, influenza, or pneumonia (ICD-9-CM 460-488) in the same calendar year and month for a length of stay ≤ 14 days and without congenital anomalies (ICD-9-CM 740-759) or prior diagnosis of child maltreatment at any point in the CDARS database. All these individuals had provided consent for future research contact at the time of study enrollment.

The follow-up research was conducted during 2018–2021. We first invited the individuals in the exposed cohort to join the follow-up assessment by phone. For every exposed individual who accepted our study invitation, one unexposed individual of the same gender, similar age of onset (varying by 1 year), similar follow-up time (varying by 1 year), and similar poverty status at the time of index hospital admission (receiving government financial assistance or not) would be invited until the matching and assessment procedures were completed.

### Ethical approval

All procedures involving human subjects in this study were approved by the Institutional Review Board of the University of Hong Kong/Hospital Authority Hong Kong Western Cluster (UW 18-442). In addition to the initial consent for future research contact, all individuals who attended the follow-up assessment were required to provide informed consent for participation in this follow-up research. For those under the age of 18 years at the time of data collection, the consent of their parent/guardian was also obtained.

### Data collection and measures

During the assessment session, the participant underwent a blood draw, performed by a phlebotomist, and completed a comprehensive set of questionnaires with items measuring their social, cognitive, and psychological conditions. Time-varying demographic characteristics including educational attainment and employment status were also collected. For participants who had difficulty completing the questionnaires by themselves such as early adolescents (less than 10% of the sample), we asked their parent/primary caregiver to complete the questionnaires on behalf of the participants.

### Social domain

The 7-item family support subscale of the Multidimensional Scale of Perceived Social Support (MSPSS) was used to assess the participant’s perceived degree of support from family members on a 7-point Likert scale, with higher scores indicating greater family support. The Chinese version of MSPSS family support subscale demonstrated excellent internal consistency (Cronbach’s alpha: 0.89) in a previous study [17].

The Parental Bonding Instrument (PBI) was used to examine the participants’ memories of their mothers and fathers during the first 16 years of life [18]. For participants under the age of 16 years, we asked them to recall the period from birth to the date of assessment. Each participant completed a scale for the mother and father, respectively. The PBI has 12 care items and 13 overprotection items on a 4-point scale, with higher scores indicating higher levels of parental care. The Chinese version of PBI has been validated in Hong Kong with good convergent validity and reliability (Cronbach’s alpha: 0.72–0.86) [19].

### Cognitive domain

Executive functioning was assessed with the Adult Executive Functioning Inventory (ADEXI) [20].The ADEXI has 14 items in two subscales: working memory and inhibition. It has been shown to have high reliability (i.e. Cronbach’s alphas around 0.90 for the ADEXI full scale) and high discriminative validity with large effect sizes (sensitivity: 0.76 and specificity: 0.91) when comparing individuals with ADHD and those without [20]. The Chinese version of ADEXI has been used in previous research studies and demonstrated good internal consistency (Cronbach’s alpha 0.80 for working memory; 0.70 for inhibition) [21].

### Psychological domain

The 10-item short version of the Connor–Davidson Resilience Scale (CD-RISC10) was used to measure the participant’s level of resilience on a five-point Likert scale ranging from 0 = not true at all to 4 = true nearly all of the time [22]. The item scores are added to a total score, with a higher score reflecting higher resilience [22]. It has been widely used in local clinical and community settings and demonstrated good validity (correlation coefficients with other psychological qualities ranging from −0.26 to 0.53) and internal consistency (Cronbach’s alpha = 0.87) [23].

The subjective and objective measurements of the participant’s stress level were conducted through the analysis of questionnaire and biomarker data. The 7-item stress scale of the Depression Anxiety Stress Scale – 21 (DASS21 stress) was used to assess the participant’s perceived stress level in the preceding week on a 4-point scale, with higher scores indicating higher levels of stress. The DASS21 stress scale has demonstrated good internal consistency (Cronbach’s alpha = 0.89) and validity (intercorrelations with the other two scales of DASS-21: 0.63 for Depression and 0.67 for Anxiety scales) [24]. In addition, inflammatory markers including IL-6, IL-10, and IL-6/IL-10 ratio were used to evaluate the physiological stress responses. Specifically, serum was extracted from the peripheral blood after 15-min centrifugation at 3000 rpm and stored at −80 °C. The serum levels of IL-6 and IL-10 were determined using multiplexed bead-based immunoassays using the LEGENDplex™ Human Inflammation Panel 1 [13-plex] kit (Biolegend, San Diego, CA). Data from the flow cytometry was then acquired using the BD LSR II System (BD Biosciences, Franklin Lakes, NJ) and analyzed by the LEGENDplex™ Data Analysis Software Suit v8.0 (Biolegend).

### Sample size

Among the 85 exposed individuals with a history of substantiated childhood maltreatment in the database, blood samples and survey data were collected from 63 individuals, whereas the remaining 22 individuals were not assessed due to invalid contact information (n = 7) or refusal to participate (n = 15). Based on the pre-specified matching criteria, the unexposed individuals were then approached and matched to the exposed individuals by a ratio of 1:1. Hence, this study analyzed 63 exposed individuals and 63 unexposed individuals. A meta-analysis found an odds ratio of 3.42 for the association between overall childhood maltreatment and non-suicidal self-injury [25]. Our assessed sample size was thus deemed adequate for detecting this medium effect size with 80% power at the 0.05 level of significance [26].

### Data analysis

After data checking for normality of distribution by means of skewness and kurtosis, descriptive statistics were presented as means and standard deviations or frequency count as appropriate. Variables with skewed distributions including IL-6, IL-10, and monthly household income at follow-up were logarithmically transformed before data analysis. Differences in demographic characteristics between exposed and unexposed individuals were assessed using the chi-square test for categorical variables and t-test for continuous variables. In addition, we performed generalized linear regression to assess the between-group differences in all measured parameters, quantified as unstandardized regression estimate (β). Two models were constructed: first with child maltreatment status as the independent variable and second with an additional adjustment for monthly household income at follow-up. For subgroup analyses, the participants were further categorized using median splits based on their age of onset (< 8 vs ≥ 8) and follow-up duration (< 14 vs ≥ 14).

**Fig. 1:**
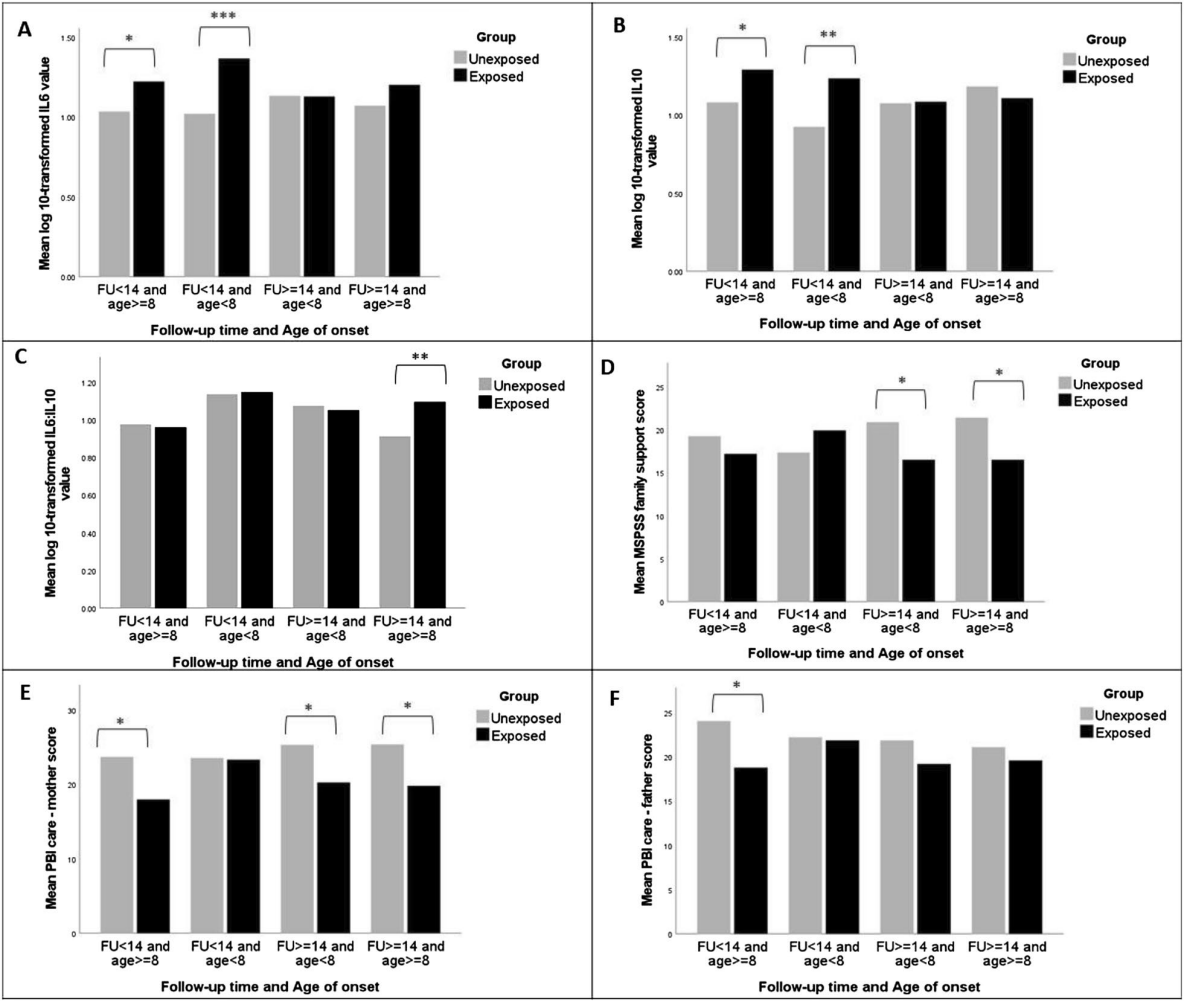
Comparison of **a** log10-transformed IL-6 value, **b** log10-transformed IL-10 value, **c** log10-transformed IL-6:IL-10 value, **d** MSPSS family support score, **e** PBI care (mother) score, and **f** PBI care (father) score between exposed and unexposed individuals stratified by follow-up time and age of onset. *p < 0.05; **p < 0.01; ***p < 0.001. Adjusted for monthly household income at follow-up

The missing data rates for the variables of interest range from 0.3% to 14.0%. Little’s MCAR X^2^[18] = 17.25, p = 0.506 indicated that the data were missing completely at random and appropriate for multiple imputation [27]. We imputed missing values using the R package mice [28]. Five data sets were imputed using predictive mean matching following 50 iterations of the algorithm. Since the imputed values were largely similar across the imputed data sets, we used the first imputed data set in this analysis. Sensitivity analyses also showed trivial differences between the original data and imputed data, so the imputed results are presented in the main results (Table 2 and Fig. 1) and complete cases in Additional file 1 results (Additional file 1: Tables S1 and S3a–d). All analyses were conducted using R version 4.1.1, with a p-value < 0.05 denoting statistical significance.

## Results

### Sample characteristics

Characteristics of exposed and unexposed individuals are shown in Table 1. All participants were followed for an average of 14.83 years and comprised more females (60.3%) than males (39.7%). Among the exposed individuals, five had repeated records of child maltreatment in the CDARS database. The unexposed individuals were confirmed with no history of childhood maltreatment by means of hospital record checking and self-report. Except for the monthly household income at follow-up, the exposed and unexposed individuals were otherwise similar with regards to poverty status on the day of hospital admission, age of onset and at follow-up, education level at follow-up, and employment status at follow-up.

**Table 1:** Subject characteristics

	Childhood maltreatment exposure		p-value
	Exposed individuals (n = 63)	Unexposed individuals (n = 63)	
Gender, n (%)			
Male	25 (39.7)	25 (39.7)	NA
Female	38 (60.3)	38 (60.3)	NA
Baseline			
Receiving governmental financial assistance on the day of hospital admission, n(%)	1 (1.6)	1 (1.6)	NA
Age of onset (years), mean(SD)	8.38 (4.76)	8.43 (4.96)	0.956
Follow-up time (years), mean(SD)	14.76 (3.07)	14.89 (2.95)	0.813
Follow-up			
Age at follow-up	23.14 (5.17)	23.32 (5.19)	0.850
Education level, n (%)			0.168
Lower secondary school or below	8 (12.7)	4 (6.3)	
Upper secondary school/Associate Degree/ Higher Diploma	38 (60.3)	33 (52.4)	
Bachelor's degree or above	17 (27.0)	26 (41.3)	
Employment status^a^, n (%)			0.690
Full-time	23 (37.1)	25 (40.3)	
Part-time	6 (9.7)	4 (6.5)	
Unemployed	4 (6.5)	7 (11.3)	
Full-time student	29 (46.8)	26 (41.9)	
Log10-transformed monthly household income, mean(SD)	4.44 (0.27)	4.60 (0.37)	0.006

^a^missing count not shown

### Outcome comparison by history of child maltreatment

Table 2 describes the between-group differences by outcome domains. Among the social variables, MSPSS family support scale scores were significantly lower in the exposed cohort than the unexposed cohort (estimated mean difference = 2.97, p = 0.010). PBI Care (mother) scale scores were also lower in the exposed cohort than the unexposed cohort (estimated mean difference = 4.64, p < 0.001). These differences remained significant after adjusting for monthly household income at follow-up. Both groups had similar scores on ADEXI executive function, CD-RISC10 resilience, and DASS21 stress scales but differed with regards to the level of log10-transformed IL-6 both before and after adjusting for monthly household income at follow-up (β = 0.15, p = 0.001). On the other hand, log10-transformed IL-10 showed significant between-group differences after adjusting for monthly household income at follow-up (β = 0.11, p = 0.011).

**Table 2:** Comparison of long-term outcomes between exposed and unexposed individuals

	Exposed individuals (n = 63)	Unexposed individuals (n = 63)	Model 1^a^		Model 2^b^	
	mean (SD)	mean (SD)	β (95% CI)	p-value	β (95% CI)	p-value
Social						
MSPSS family support	17.27 (6.55)	20.24 (6.52)	−2.97 (−5.23, −0.70)	0.010	−2.48 (−4.79, −0.17)	0.035
PBI care (mother)	20.05 (7.14)	24.68 (6.52)	−4.64 (−7.00, −2.27)	< 0.001	−4.19 (−6.62, −1.77)	0.001
PBI overprotection (mother)	14.22 (6.97)	13.57 (6.99)	0.65 (−1.77, 3.07)	0.598	0.60 (−1.90, 3.09)	0.639
PBI care (father)	19.63 (8.05)	22.22 (7.26)	−2.59 (−5.24, 0.07)	0.056	−1.83 (−4.51, 0.86)	0.182
PBI overprotection (father)	12.05 (7.06)	10.48 (7.15)	1.57 (−0.89, 4.03)	0.211	1.57 (−0.97, 4.11)	0.226
Cognitive						
ADEXI working memory	21.86 (6.15)	22.22 (6.29)	−0.37 (−2.52, 1.79)	0.740	−0.54 (−2.76, 1.68)	0.634
ADEXI inhibition	13.65 (3.44)	13.44 (3.59)	0.21 (−1.01, 1.42)	0.740	0.07 (−1.18, 1.32)	0.913
Psychological						
CD-RISC10 resilience	30.83 (5.56)	30.06 (5.79)	0.76 (−1.20, 2.73)	0.447	0.87 (−1.16, 2.90)	0.401
DASS21 stress	24.48 (6.63)	24.73 (6.20)	−0.25 (−2.48, 1.97)	0.823	−0.45 (−2.74, 1.84)	0.699
Log10-transformed IL-6	1.21 (0.29)	1.08 (0.23)	0.14 (0.05, 0.23)	0.003	0.15 (0.06, 0.24)	0.001
Log10-transformed IL-10	1.17 (0.25)	1.09 (0.24)	0.08 (−0.01, 0.16)	0.076	0.11 (0.03, 0.19)	0.011
Log10-transformed IL-6:IL-10	1.06 (0.23)	1.00 (0.20)	0.05 (−0.03, 0.13)	0.202	−0.10 (−0.22, 0.02)	0.095

^a^With exposed individuals/unexposed individuals (reference group) as the independent variable

^b^Model 1 adjusted for monthly household income at follow-up

### Subgroup analysis by age of onset and follow-up duration

Based on the whole group results, we further compared the selected variables, namely MSPSS family support, PBI Care (mother), PBI Care (father), log10-transformed IL-6, and log10-transformed IL-10, among subgroups stratified by age of onset (< 8 vs ≥ 8) and follow-up duration (< 14 vs ≥ 14) (Fig. 1). The results of univariate and multivariate analyses were provided in Additional file 1: Tables S2a–d and highlighted below:

Regarding the social variables, MSPSS family support and PBI Care (mother) scale scores showed significant between-group differences when the analyses were restricted to those with FU ≥ 14 and age < 8 (family support: β = −4.36, p = 0.020; maternal care: β = −5.05, p = 0.026) and those with FU ≥ 14 and age ≥ 8 (family support: β = −4.89, p = 0.021; maternal care: β = −5.56, p = 0.010). These differences remained significant after adjusting for monthly household income at follow-up. When the analyses were restricted to those with FU < 14 and age ≥ 8, although univariate analyses showed significant between-group differences in both PBI Care (mother) and PBI Care (father) scale scores, these differences became trival after adjusting for monthly household income at follow-up.

Regarding the stress-related biomarkers, there were significant between-group differences in log10-transformed IL-6 and log10-transformed IL-10 values when the analyses were restricted to those with FU < 14 and age ≥ 8 (log10-transformed IL-6: β = 0.19, p = 0.029; log10-transformed IL-10: β = 0.21, p = 0.013) and those with FU < 14 and age < 8 (log10-transformed IL-6: β = 0.35, p < 0.001; log10-transformed IL-10: β = 0.31, p = 0.001). On the other hand, the between-group differences in log10-transformed ratio of IL-6 to IL-10 values were significant only when the analyses were restricted to those with FU ≥ 14 and age ≥ 8 (β = 0.18, p = 0.003). These differences remained statistically significant in the adjusted model.

## Discussion

Findings from the current study offer new insights into the long-term consequences of child maltreatment. By investigating a wide range of functioning outcomes, we found that a history of substantiated childhood maltreatment was associated with later memories of inadequate maternal care during childhood. It also predicted lower perceived family support and higher IL-6 and IL-10 levels later in life. The findings echo with previous findings of the associations between child maltreatment and cytokines profile in clinical samples [8, 9]. In addition, we noticed that the exposed individuals had slightly poorer working memory than the unexposed individuals. Compared with the unexposed individuals, a smaller proportion of exposed individuals were able to complete tertiary education and have full-time employment at the follow-up assessment. These findings indicate the need of prevocational skills training and on-the-job coaching for individuals with a history of childhood maltreatment. In addition, the exposed individuals reported slightly higher levels of resilience and lower levels of perceived stress than the unexposed individuals, which is consistent with previous research showing that high resilience can buffer against emotional problems following childhood adversity [29, 30]. Given the complex nature of resilience, future research should examine how to promote resilience in the general population before the occurrence of adverse events.

Considering the call for more child maltreatment research in non-Western societies [7] and the very few studies with long follow-up intervals [4], the present study investigated the long-term consequences of childhood maltreatment with a follow-up period up to 20 years in a Chinese society. The findings can contribute to a better understanding of the effect of child maltreatment in non-Western cultures. A previous study in the United States found that perceived social support from family predicted lower trauma symptoms across all levels of maltreatment for men, whereas this protective effect diminished as the severity of maltreatment increased for women [31]. Consistent with this previous research, we found an association between childhood maltreatment experiences and the perception of poor family support in adulthood. Interventions should be designed to strengthen family relationships in child maltreatment victims across stages of development.

Notably, the finding of significant between-group differences in perceived care from mothers but not fathers suggests that the occurrence of child maltreatment may relate more to maternal care than paternal care, which echoes previous research documenting differences in child behavior management techniques between mothers and fathers [32]. In addition to the differences in parental care and family support, significant between-group differences were found in physiological stress responses, albeit no differences in self-reported psychological qualities and cognitive functions. These findings demonstrate the need for research using both questionnaires and biomarkers to gain a more complete understanding of the long-term consequences of childhood adversity. Previous studies have documented higher inflammatory stress responses in individuals with lower socioeconomic status [33] and those with higher levels of anger responses during stress exposure, in combination with less social support [34]. Consistent with these findings, this study observed higher levels of inflammatory markers, both IL-6 and IL-10, among individuals with a history of childhood maltreatment than those without the exposure, suggesting that child maltreatment victims may suffer from low-grade systemic inflammation and are at an increased risk for diseases [35].

Further analyses stratified by age of onset and follow-up duration showed that older victims tended to perceive poorer maternal care during childhood and lower family support later in life. The results may be explained by the notion of intergenerational transmission of social disadvantage [36], wherein insufficient or inferior resources in the parental generation (e.g. poor perceived maternal care in childhood) could be replicated in the second generation (e.g. poor perceived family support in adulthood). Another finding of note is that only individuals exposed to the event at age 8 or above with a follow-up period of less than 14 years demonstrated significant between-group differences in the recall of both maternal and paternal care. These finding suggests that the memories of parental care are particularly strong in the first decade following the adverse event, particularly for those individuals with competent cognitive skills at the time of exposure to the trauma.

On the other hand, the significant between-group differences in IL-6 and IL-10 levels were found only when the follow-up duration was less than 14 years. This finding is consistent with a previous study demonstrating an association between childhood bullying and low-grade systemic inflammation into young adulthood [37]. Furthermore, we found that among individuals aged 22 years or above, a history of childhood maltreatment was associated with a higher ratio of IL-6:IL-10. An emerging body of research has demonstrated that depressive-like behavior may result from concurrent increases in IL-6 and decreases in IL-10 [38]. Further studies with bigger samples should explore whether memories of traumatic events will interact with trait rumination to affect the proinflammatory and anti-inflammatory cytokine profiles among individuals with depressive disorders.

This study has several limitations that should be considered when interpreting the results. First, the study sample was small and recruited from a teaching hospital in Hong Kong, which limits the generalizability of results to a broader context. Second, although we studied the timing of exposure, we did not explore the effect of maltreatment type and severity due to the lack of relevant data coupled with small sample size. Future studies should examine the effect of maltreatment type and timing on long-term health and development. For example, maltreatment recurrence may strengthen the association between childhood adversity and health problems. Third, this study measured executive functions through questionnaires which may involve biases. Future research should use objective assessments such as computerized tests to measure attentional control and working memory. In addition, while the unexposed individuals were confirmed to have no maltreatment exposure prior to the follow-up assessment, recent life changes such as job loss or relocation may influence the stress level and inflammatory responses. Future research should collect data on these events and treat them as covariates in the analyses.

## Conclusion

Childhood maltreatment is known to have adverse effects on long-term health and well-being. This study provides additional evidence illustrating the long-term outcomes of child maltreatment in a non-Western society. Findings suggest that the manifestation of diseases following childhood maltreatment events may depend on the timing of exposure to the trauma and period of assessment. Effective interventions for child maltreatment victims should be tailored according to the nature of their trauma exposure and reviewed regularly to address their health and psychosocial concerns at different life stages.

## Supplementary Information

**Additional file 1: Table S1.** Comparison of long-term outcomes between exposed and unexposed individuals (based on original data). **Table S2.** Comparison of long-term outcomes between exposed and unexposed individuals (based on imputed data) stratified by follow-up time and age of onset**Table S3.**Comparison of long-term outcomes between exposed and unexposed individuals (based on original data) stratified by follow-up time and age of onset

## Data Availability

The data that support the findings of this study are available on request from the corresponding author.
