# Supplementary material for: Evidence of individual differences in the long-term social, psychological, and cognitive consequences of child maltreatment
Source: Child Adolesc Psychiatry Ment Health. 2022 Nov 23;16:88. doi: 10.1186/s13034-022-00524-4 (PMC9686092; doi:10.1186/s13034-022-00524-4)
Supplement: Supplementary file 1 — Additional file 1: Table S1. Comparison of long-term outcomes between exposed and unexposed individuals (based on original data). Table S2. Comparison of long-term outcomes between exposed and unexposed individuals (based on imputed data) stratified by follow-up time and age of onsetTable S3.Comparison of long-term outcomes between exposed and unexposed individuals (based on original data) stratified by follow-up time and age of onset [file 13034_2022_524_MOESM1_ESM.docx]

Supplementary table 1. Comparison of long-term outcomes between exposed and unexposed individuals (based on original data)

|  | Exposed individuals | Unexposed individuals | Model 1^a^ | | Model 2^b^ | |
| --- | --- | --- | --- | --- | --- | --- |
|  | mean(SD) | mean(SD) | β (95%CI) | p-value | β (95%CI) | p-value |
| ***Social*** |  |  |  |  |  |  |
| MSPSS family support | 17.32(6.59) | 20.24(6.52) | -2.92(-5.20,-0.64) | 0.012 | -2.73(-5.08,-0.38) | 0.023 |
| PBI Care (mother) | 20.07(7.38) | 24.69(6.70) | -4.63(-7.17,-2.08) | <0.001 | -4.32(-6.97,-1.67) | 0.001 |
| PBI Overprotection (mother) | 14.07(6.8) | 13.72(6.67) | 0.35(-2.10,2.79) | 0.781 | 0.57(-1.97,3.11) | 0.662 |
| PBI Care (father) | 19.71(7.71) | 22.63(6.95) | -2.91(-5.66,-0.17) | 0.037 | -2.67(-5.50,0.16) | 0.064 |
| PBI Overprotection (father) | 10.67(6.28) | 10.4(7.06) | 0.27(-2.26,2.80) | 0.836 | 0.46(-2.18,3.10) | 0.734 |
| ***Cognitive*** |  |  |  |  |  |  |
| ADEXI working memory | 21.84(6.35) | 22.22(6.29) | -0.38(-2.64,1.87) | 0.739 | -0.43(-2.78,1.91) | 0.718 |
| ADEXI inhibition | 13.54(3.52) | 13.44(3.59) | 0.10(-1.16,1.35) | 0.878 | -0.004(-1.31,1.30) | 0.995 |
| ***Psychological*** |  |  |  |  |  |  |
| CD-RISC10  resilience | 30.85(5.6) | 30.06(5.79) | 0.79(-1.19,2.77) | 0.434 | 0.46(-1.56,2.48) | 0.656 |
| DASS21 stress | 24.57(6.7) | 24.85(6.24) | -0.29(-2.57,2.00) | 0.807 | -0.32(-2.74,2.10) | 0.795 |
| Log10-transformed IL-6 | 1.08(0.24) | 1.01(0.23) | 0.06(-0.02,0.15) | 0.147 | 0.07(-0.02,0.16) | 0.136 |
| Log10-transformed IL-10 | 1.09(0.29) | 1.05(0.28) | 0.04(-0.07,0.14) | 0.512 | 0.07(-0.03,0.17) | 0.192 |
| Log10-transformed IL-6:IL-10 | 1.02(0.25) | 1.01(0.24) | 0.01(-0.08,0.10) | 0.837 | -0.01(-0.10,0.09) | 0.924 |
| ^a^ With exposed individuals/unexposed individuals (reference group) as the independent variable | | | | | | |
| ^b^ Model 1 adjusted for monthly household income at follow-up | | | | | | |

Supplementary table 2. Comparison of long-term outcomes between exposed and unexposed individuals (based on imputed data) stratified by follow-up time and age of onset

(a) Follow-up time < 14 and age ≥ 8

|  | Exposed individuals  (n=16) | Unexposed individuals (n=17) | Model 1^a^ | | Model 2^b^ | |
| --- | --- | --- | --- | --- | --- | --- |
|  | mean(SD) | mean(SD) | β (95%CI) | p-value | β (95%CI) | p-value |
| MSPSS family support | 17.19(6.66) | 19.24(7.75) | -2.05(-6.84,2.74) | 0.402 | -1.16(-6.02,3.71) | 0.641 |
| PBI Care (mother) | 17.94(7.71) | 23.65(7.23) | -5.71(-10.65,-0.77) | 0.023 | -4.34(-9.19,0.51) | 0.080 |
| PBI Care (father) | 18.75(8.58) | 24.00(5.27) | -5.25(-9.93,-0.57) | 0.028 | -4.50(-9.27,0.28) | 0.065 |
| Log10-transformed IL-6 | 1.22(0.22) | 1.03(0.28) | 0.19(0.02,0.36) | 0.029 | 0.19(0.02,0.37) | 0.030 |
| Log10-transformed IL-10 | 1.29(0.22) | 1.08(0.27) | 0.21(0.05,0.37) | 0.013 | 0.26(0.10,0.42) | 0.001 |
| Log10-transformed IL-6:IL-10 | 0.96(0.17) | 0.98(0.21) | -0.02(-0.14,0.11) | 0.798 | -0.05(-0.18,0.07) | 0.415 |
| ^a^ With exposed individuals/unexposed individuals (reference group) as the independent variable | | | | | | |
| ^b^ Model 1 adjusted for monthly household income at follow-up | | | | | | |

(b) Follow-up time < 14 and age < 8

|  | Exposed individuals (n=11) | Unexposed individuals (n=6) | Model 1^a^ | | Model 2^b^ | |
| --- | --- | --- | --- | --- | --- | --- |
|  | mean(SD) | mean(SD) | β (95%CI) | p-value | β (95%CI) | p-value |
| MSPSS family support | 19.91(6.5) | 17.33(6.31) | 2.58(-3.44,8.59) | 0.402 | 1.85(-3.80,7.50) | 0.521 |
| PBI Care (mother) | 23.27(3.55) | 23.5(5.65) | -0.23(-4.31,3.85) | 0.913 | -0.72(-4.55,3.11) | 0.712 |
| PBI Care (father) | 21.82(6.42) | 22.17(6.05) | -0.35(-6.23,5.53) | 0.908 | -1.02(-6.59,4.55) | 0.721 |
| Log10-transformed IL-6 | 1.37(0.23) | 1.02(0.12) | 0.35(0.16,0.53) | <0.001 | 0.36(0.18,0.54) | <0.001 |
| Log10-transformed IL-10 | 1.23(0.21) | 0.93(0.14) | 0.31(0.13,0.49) | 0.001 | 0.29(0.12,0.45) | 0.001 |
| Log10-transformed IL-6:IL-10 | 1.15(0.35) | 1.13(0.26) | 0.01(-0.29,0.31) | 0.944 | 0.05(-0.23,0.33) | 0.732 |
| ^a^ With exposed individuals/unexposed individuals (reference group) as the independent variable | | | | | | |
| ^b^ Model 1 adjusted for monthly household income at follow-up | | | | | | |

(c) Follow-up time ≥ 14 and age < 8

|  | Exposed individuals (n=18) | Unexposed individuals (n=22) | Model 1^a^ | | Model 2^b^ | |
| --- | --- | --- | --- | --- | --- | --- |
|  | mean(SD) | mean(SD) | β (95%CI) | p-value | β (95%CI) | p-value |
| MSPSS family support | 16.50(5.76) | 20.86(6.32) | -4.36(-8.05,-0.67) | 0.020 | -4.30(-8.34,-0.27) | 0.037 |
| PBI Care (mother) | 20.22(8.36) | 25.27(6.32) | -5.05(-9.48,-0.62) | 0.026 | -4.89(-9.73,-0.04) | 0.048 |
| PBI Care (father) | 19.17(8.06) | 21.82(7.59) | -2.65(-7.39,2.09) | 0.273 | -1.17(-6.22,3.89) | 0.651 |
| Log10-transformed IL-6 | 1.13(0.30) | 1.13(0.21) | -0.004(-0.16,0.15) | 0.958 | 0.03(-0.14,0.20) | 0.736 |
| Log10-transformed IL-10 | 1.09(0.24) | 1.08(0.25) | 0.01(-0.14,0.16) | 0.895 | 0.05(-0.11,0.21) | 0.504 |
| Log10-transformed IL-6:IL-10 | 1.05(0.20) | 1.07(0.18) | -0.02(-0.14,0.09) | 0.680 | -0.03(-0.16,0.09) | 0.587 |
| ^a^ With exposed individuals/unexposed individuals (reference group) as the independent variable | | | | | | |
| ^b^ Model 1 adjusted for monthly household income at follow-up | | | | | | |

(d) Follow-up time ≥ 14 and age ≥ 8

|  | Exposed individuals (n=18) | Unexposed individuals (n=18) | Model 1^a^ | | Model 2^b^ | |
| --- | --- | --- | --- | --- | --- | --- |
|  | mean(SD) | mean(SD) | β (95%CI) | p-value | β (95%CI) | p-value |
| MSPSS family support | 16.5(7.33) | 21.39(5.62) | -4.89(-9.03,-0.74) | 0.021 | -4.71(-8.93,-0.48) | 0.029 |
| PBI Care (mother) | 19.78(6.73) | 25.33(6.68) | -5.56(-9.81,-1.30) | 0.010 | -5.43(-9.77,-1.08) | 0.014 |
| PBI Care (father) | 19.56(8.84) | 21.06(8.91) | -1.50(-7.13,4.13) | 0.602 | -0.95(-6.64,4.75) | 0.745 |
| Log10-transformed IL-6 | 1.20(0.34) | 1.07(0.23) | 0.13(-0.05,0.32) | 0.167 | 0.16(-0.03,0.34) | 0.092 |
| Log10-transformed IL-10 | 1.11(0.26) | 1.18(0.2) | -0.08(-0.22,0.08) | 0.327 | -0.07(-0.22,0.08) | 0.381 |
| Log10-transformed IL-6:IL-10 | 1.09(0.21) | 0.91(0.16) | 0.18(0.06,0.30) | 0.003 | 0.20(0.09,0.32) | 0.001 |
| ^a^ With exposed patients/unexposed patients (reference group) as the independent variable | | | | | | |
| ^b^ Model 1 adjusted for monthly household income at follow-up | | | | | | |

Supplementary table 3. Comparison of long-term outcomes between exposed and unexposed individuals (based on original data) stratified by follow-up time and age of onset

(a) Follow-up time < 14 and age ≥ 8

|  | Exposed individuals | Unexposed individuals | Model 1^a^ | | Model 2^b^ | |
| --- | --- | --- | --- | --- | --- | --- |
|  | mean(SD) | mean(SD) | β (95%CI) | p-value | β (95%CI) | p-value |
| MSPSS family support | 17.40(6.83) | 19.24(7.75) | -1.88(-6.98,3.23) | 0.471 | -1.22(-6.33,3.88) | 0.639 |
| PBI Care (mother) | 17.60(7.85) | 23.20(7.60) | -5.49(-11.01,0.04) | 0.052 | -4.00(-9.43,1.42) | 0.148 |
| PBI Care (father) | 19.27(8.62) | 24.00(5.27) | -5.21(-10.03,-0.40) | 0.034 | -4.84(-9.71,0.02) | 0.051 |
| Log10-transformed IL-6 | 1.17(0.24) | 0.93(0.23) | 0.22(0.05,0.39) | 0.011 | 0.20(0.03,0.37) | 0.020 |
| Log10-transformed IL-10 | 1.29(0.33) | 1.04(0.29) | 0.19(-0.01,0.39) | 0.056 | 0.25(0.06,0.44) | 0.011 |
| Log10-transformed IL-6:IL-10 | 0.91(0.2) | 0.97(0.26) | -0.05(-0.22,0.12) | 0.593 | -0.08(-0.25,0.08) | 0.321 |
| ^a^ With exposed individuals/unexposed individuals (reference group) as the independent variable | | | | | | |
| ^b^ Model 1 adjusted for monthly household income at follow-up | | | | | | |

(b) Follow-up time < 14 and age < 8

|  | Exposed individuals | Unexposed individuals | Model 1^a^ | | Model 2^b^ | |
| --- | --- | --- | --- | --- | --- | --- |
|  | mean(SD) | mean(SD) | β (95%CI) | p-value | β (95%CI) | p-value |
| MSPSS family support | 19.91(6.50) | 17.33(6.31) | 2.37(-3.92,8.65) | 0.461 | 1.88(-4.00,7.77) | 0.530 |
| PBI Care (mother) | 23.56(3.91) | 23.50(5.65) | -0.25(-4.94,4.44) | 0.917 | -0.66(-5.12,3.80) | 0.772 |
| PBI Care (father) | 21.22(5.93) | 22.20(6.76) | -1.20(-7.85,5.45) | 0.724 | -2.43(-8.53,3.66) | 0.434 |
| Log10-transformed IL-6 | 1.28(0.21) | 0.97(0.14) | 0.33(0.14,0.51) | 0.001 | 0.31(0.12,0.51) | 0.001 |
| Log10-transformed IL-10 | 1.16(0.21) | 0.87(0.16) | 0.29(0.09,0.48) | 0.005 | 0.26(0.10,0.42) | 0.002 |
| Log10-transformed IL-6:IL-10 | 1.11(0.32) | 1.17(0.31) | -0.04(-0.37,0.29) | 0.808 | -0.01(-0.34,0.32) | 0.965 |
| ^a^ With exposed patients/unexposed individuals (reference group) as the independent variable | | | | | | |
| ^b^ Model 1 adjusted for monthly household income at follow-up | | | | | | |

(c) Follow-up time ≥ 14 and age < 8

|  | Exposed individuals | Unexposed individuals | Model 1^a^ | | Model 2^b^ | |
| --- | --- | --- | --- | --- | --- | --- |
|  | mean(SD) | mean(SD) | β (95%CI) | p-value | β (95%CI) | p-value |
| MSPSS family support | 16.5(5.76) | 20.86(6.32) | -4.36(-8.05,-0.67) | 0.020 | -4.30(-8.34,-0.27) | 0.037 |
| PBI Care (mother) | 20.35(8.59) | 25.60(6.55) | -5.25(-10.00,-0.50) | 0.030 | -4.99(-10.25,0.27) | 0.063 |
| PBI Care (father) | 19.54(8.3) | 21.86(7.77) | -2.32(-7.67,3.03) | 0.396 | -0.95(-6.57,4.68) | 0.742 |
| Log10-transformed IL-6 | 0.94(0.18) | 1.09(0.23) | -0.15(-0.29,-0.02) | 0.029 | -0.13(-0.28,0.02) | 0.083 |
| Log10-transformed IL-10 | 0.96(0.23) | 1.05(0.32) | -0.09(-0.28,0.10) | 0.338 | -0.03(-0.23,0.18) | 0.799 |
| Log10-transformed IL-6:IL-10 | 1.02(0.24) | 1.08(0.22) | -0.06(-0.21,0.09) | 0.413 | -0.09(-0.25,0.07) | 0.253 |
| ^a^ With exposed individuals/unexposed individuals (reference group) as the independent variable | | | | | | |
| ^b^ Model 1 adjusted for monthly household income at follow-up | | | | | | |

(d) Follow-up time ≥ 14 and age ≥ 8

|  | Exposed individuals | Unexposed individuals | Model 1^a^ | | Model 2^b^ | |
| --- | --- | --- | --- | --- | --- | --- |
|  | mean(SD) | mean(SD) | β (95%CI) | p-value | β (95%CI) | p-value |
| MSPSS family support | 16.50(7.33) | 21.39(5.62) | -5.57(-9.62,-1.51) | 0.007 | -5.34(-9.47,1.23) | 0.011 |
| PBI Care (mother) | 20.13(6.73) | 25.33(6.68) | -5.47(-9.96,-0.97) | 0.017 | -5.43(-9.96,-0.89) | 0.019 |
| PBI Care (father) | 19.33(7.85) | 22.31(7.84) | -3.31(-9.20,2.57) | 0.270 | -3.12(-9.06,2.83) | 0.304 |
| Log10-transformed IL-6 | 1.00(0.20) | 1.00(0.24) | -0.10(-0.16,0.15) | 0.949 | 0.03(-0.12,0.17) | 0.727 |
| Log10-transformed IL-10 | 0.98(0.25) | 1.13(0.22) | -0.15(-0.32,0.030) | 0.093 | -0.12(-0.29,0.05) | 0.167 |
| Log10-transformed IL-6:IL-10 | 1.06(0.24) | 0.88(0.17) | 0.17(0.02,0.31) | 0.026 | 0.19(0.05,0.34) | 0.007 |
| ^a^ With exposed individuals/unexposed individuals (reference group) as the independent variable | | | | | | |
| ^b^ Model 1 adjusted for monthly household income at follow-up | | | | | | |
